# Sequence-indexed mutations in maize using the UniformMu transposon-tagging population

**DOI:** 10.1186/1471-2164-8-116

**Published:** 2007-05-09

**Authors:** A Mark Settles, David R Holding, Bao Cai Tan, Susan P Latshaw, Juan Liu, Masaharu Suzuki, Li Li, Brent A O'Brien, Diego S Fajardo, Ewa Wroclawska, Chi-Wah Tseung, Jinsheng Lai, Charles T Hunter, Wayne T Avigne, John Baier, Joachim Messing, L Curtis Hannah, Karen E Koch, Philip W Becraft, Brian A Larkins, Donald R McCarty

**Affiliations:** 1 Horticultural Sciences Department, University of Florida, Gainesville, FL 32611, USA; 2 Department of Plant Sciences, University of Arizona, Tucson, AZ 85721, USA; 3 Department of Genetics, Development and Cell Biology, Iowa State University, Ames, IA 50011, USA; 4 Waksman Institute, Rutgers University, Piscataway, NJ 08854, USA

## Abstract

**Background:**

Gene knockouts are a critical resource for functional genomics. In Arabidopsis, comprehensive knockout collections were generated by amplifying and sequencing genomic DNA flanking insertion mutants. These Flanking Sequence Tags (FSTs) map each mutant to a specific locus within the genome. In maize, FSTs have been generated using DNA transposons. Transposable elements can generate unstable insertions that are difficult to analyze for simple knockout phenotypes. Transposons can also generate somatic insertions that fail to segregate in subsequent generations.

**Results:**

Transposon insertion sites from 106 UniformMu FSTs were tested for inheritance by locus-specific PCR. We confirmed 89% of the FSTs to be germinal transposon insertions. We found no evidence for somatic insertions within the 11% of insertion sites that were not confirmed. Instead, this subset of insertion sites had errors in locus-specific primer design due to incomplete or low-quality genomic sequences. The locus-specific PCR assays identified a knockout of a 6-phosphogluconate dehydrogenase gene that co-segregates with a seed mutant phenotype. The mutant phenotype linked to this knockout generates novel hypotheses about the role for the plastid-localized oxidative pentose phosphate pathway during grain-fill.

**Conclusion:**

We show that FSTs from the UniformMu population identify stable, germinal insertion sites in maize. Moreover, we show that these sequence-indexed mutations can be readily used for reverse genetic analysis. We conclude from these data that the current collection of 1,882 non-redundant insertion sites from UniformMu provide a genome-wide resource for reverse genetics.

## Background

An important aspect of functional genomics is understanding the phenotypic consequence of mutations in all genes in a genome. A comprehensive collection of gene knockouts allows a defined set of mutations to be systematically studied for more efficient association of genes to functions (reviewed in [1]). Multiple approaches have been used to develop comprehensive knockout resources. Biological differences between organisms make specific technologies such as homologous recombination, RNA interference, or insertional mutagenesis more useful in generating a resource for an individual species. In plants, a comprehensive knockout collection was generated for *Arabidopsis thaliana *via mutagenesis with insertion tags [2-5]. Genomic DNA flanking each tag was systematically amplified and sequenced from each mutant. These Flanking Sequence Tags (FSTs) index each mutant to the genome and are accessible through the SIGnAL T-DNA Express database, which links the mutant stocks to genome annotations [6]. A similar FST approach was used to develop a rice functional genomics resource based on insertional mutagenesis populations [7-10]. The current rice collections have more than 140,000 insertion lines with associated FSTs that are integrated at the OryGenesDB database [11].

Maize is comparable to rice as a model grass species for genome studies. Similar to rice, the maize genome is now being sequenced with a minimal tiling path strategy [12]. There are also extensive gene-enriched sequences that are estimated to include partial sequence from 95% of maize genes with smaller introns [12,13]. In contrast to rice, maize is a monoecious plant, and maize has a shorter life cycle. These biological characteristics facilitate genetic analysis. Also, maize inbreds are highly polymorphic. Robust PCR markers, genetic maps, and recombinant inbred lines have been developed that aid quantitative trait studies and positional cloning (reviewed in [14]). A comprehensive knockout collection of maize mutants would complement the existing genome resources to make functional genomics studies in maize simple and rapid.

There are multiple insertion-tagged maize populations that were generated with either *Activator (Ac) *or *Mutator *(*Mu*) transposons (reviewed in [15]). There are >150,000 mutagenized lines among the combined *Mu *populations [16-20]. These *Mu *lines are expected to have more than 1.5 million independent insertions, because *Mu *transposons accumulate to high copy numbers within individual plants (reviewed in [21]). Moreover, *Mu *elements show a bias for inserting into or near transcribed regions of the genome and are associated with a high rate of forward mutagenesis [17,19,22,23]. This high mutation frequency makes *Mu *mutagenesis attractive for generating knockout resources, but the high-copy nature of *Mu *elements presents a challenge in isolating individual insertion sites for sequencing. Single plants have multiple germinal insertions that represent both progenitor mutations and mutations unique to the individual. In addition, active transposon systems can generate somatic insertions that fail to segregate in subsequent generations. Due to these challenges, most *Mu *populations have been developed to conduct reverse genetics screens for only one gene at a time [16,18,20].

FSTs have been generated from two *Mu *populations. Fernandes et al. [17] identified 14,887 non-redundant FSTs using a transgenic *Mu1 *element that was engineered for plasmid rescue of genomic flanking sequences. The plasmids were isolated from pools of actively transposing plants and many of the FSTs are from somatic insertions. A key challenge to sequencing FSTs from pools of actively transposing plants is identifying germinal insertion sites and associating the germinal insertions to individual seed stocks. Fernandes et al. [17] sequenced from two dimensional grids. Recovery of the same FST from both row and column pools of plants was used to associate 528 of the plasmid-rescue insertion sites to individual plants.

In contrast, FSTs from the UniformMu population were generated with both a strategy to associate each FST to individual lines and to select for germinal insertions [19,23]. UniformMu is a *Mu *population that is introgressed into the color-converted W22 inbred. Previously, we showed that UniformMu is a robust forward genetics resource for identifying visible mutant phenotypes [19]. UniformMu FSTs were isolated by amplifying native transposon insertion sites with a modified TAIL-PCR protocol, MuTAIL [23]. MuTAIL products were cloned from individual lines into small libraries. Sequencing randomly selected clones, similar to an EST strategy, recovers 67%–75% of the insertion sites from each line. With this sequencing strategy, each FST corresponds to an individual plant or maize ear.

The UniformMu FSTs also derive from plants in which the *Mu *transposons were stabilized by selecting against somatic transposition using the *bronze1-mum9 *(*bz1-mum9*) mutation [24,25]. The selection against somatic activity predicts that all of the FSTs should be from germinal insertion sites. Database searches with 1,737 non-redundant UniformMu FSTs indicated a bias for insertions in or near transcribed regions of the genome[19]. Moreover, *in silico *mapping of the FSTs indicated that UniformMu transposon insertions are distributed randomly throughout the maize chromosomes suggesting the transposon mutagenesis gave rise to mutations on a genome-wide basis. These properties predict that UniformMu FSTs will be a resource of sequence-indexed mutations that is simple to use for reverse genetics applications.

Here we test the utility of UniformMu FSTs for reverse genetics. We assayed a sample of FSTs for germinal segregation. We focused on FSTs that were predominantly recovered from individual UniformMu lines. FSTs found only in one plant have the highest possibility of representing somatic insertions and provide the strongest test for whether somatic insertions exist within the UniformMu FSTs. Our data indicate that 89% of the UniformMu FSTs can be confirmed as germinal insertions by designing only one or two locus-specific primers for PCR validation. The most common problems for the 11% of insertions that were not validated as germinal insertions were design errors in the locus-specific primers due to low quality sequence data or human error. One of the FSTs co-segregates with a seed mutant that suggests an important role for chloroplast-localized oxidative pentose phosphate pathway enzymes in grain-fill, embryogenesis, and plant development. These results demonstrate that the strategy for sequencing UniformMu FSTs creates effective reverse genetics resources for characterizing gene knockouts in maize.

## Results

A sample of 106 sequence-indexed insertion sites from the UniformMu population were tested for germinal inheritance. The UniformMu FSTs represent novel insertion sites from the individual lines as well as progenitor sequences that are found throughout the population. The FSTs initially were clustered by both blastcluster analysis [19] and CAP3 assembly [26]to identify non-redundant sequences. A total of 1,434 FSTs unique to individual UniformMu seed mutants and 283 FSTs found in two to three mutants were identified with these clustering methods. We selected 79 FSTs found in individual mutants, 19 FSTs found in two mutants, and 8 FSTs found in multiple UniformMu lines for PCR validation. The 98 low redundancy insertions found in 1–2 mutants were recovered from 50 of the lines analyzed by MuTAIL sequencing. Thus, the 106 insertions sites tested were a significant sample of both UniformMu lines and MuTAIL FSTs.

**Figure 1:**
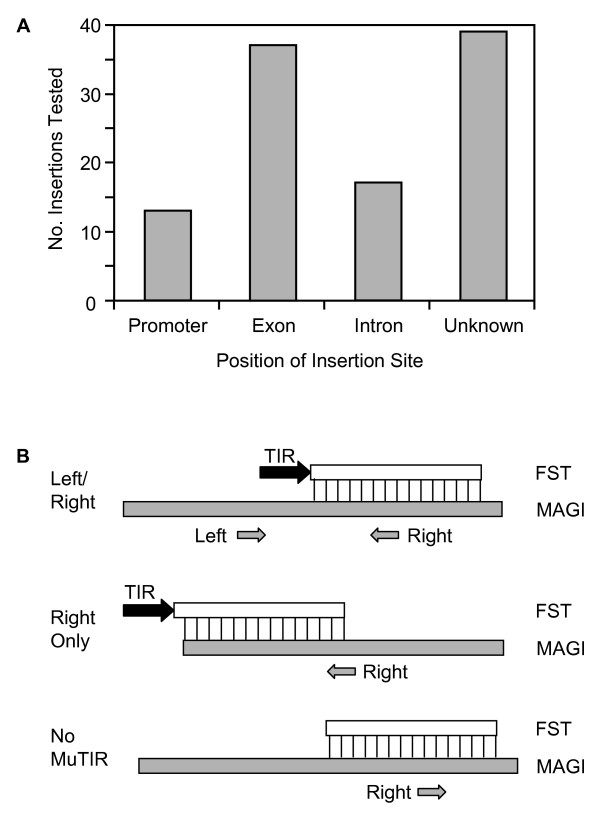
**Informatic analysis of UniformMu FSTs validated by PCR**. (**A**) Distribution of *Mu *insertion sites relative to transcribed maize sequences. (**B**) Schematics of the locus-specific primer design as constrained by available sequences. Left and right specific primers were designed when genomic sequence was available on either side of the insertion site. Right specific primers were designed when the insertion site defined novel maize genomic sequence. A presumptive right primer was designed for FSTs without a MuTIR sequence.

The FSTs selected for PCR validation were compared to the maize assembled genomic islands (MAGI) database as well as the TIGR *Zea mays *Gene Index (ZMGI) to determine the position and identity of the insertion sites [27,28]. Both the MAGI and ZMGI databases are enriched for maize gene sequences. The insertions were categorized as being within exons, introns, promoters, or unknown sequences (Fig 1A). Sixty-three percent of the insertion sites are found within 850 bp of transcribed sequences as defined by either a match of greater than 95% sequence identity between the FST and a ZMGI sequence or a match to a MAGI sequence that shows sequence identity to a ZMGI sequence (see Table 1 and Additional file 1). The remaining 39 FSTs either matched 1–2 MAGI sequences that contained no EST support for a transcribed sequence or did not show a significant match to MAGI or ZMGI sequences.

The FSTs were also analyzed for the presence of the conserved *Mu *Terminal Inverted Repeat (MuTIR). MuTAIL-PCR products are amplified with 29 bp of MuTIR sequence downstream of the transposon specific primer [23]. Presence of the MuTIR sequence identifies the precise insertion site for a FST (see Table 1 and Additional file 1). MuTIR sequences were present in 91 FSTs and absent in the remaining 15 insertions sites selected for validation. MuTIR sequences are expected to be absent from a proportion of FSTs, because the MuTAIL products were cloned in random orientations and are frequently larger than single-pass sequence reads. Locus-specific PCR primers were designed based on the location of the MuTIR and MAGI sequence matches. We used three strategies to design the primers (Fig 1B). First, left and/or right primers were designed when the precise insertion site was placed within a MAGI sequence. The left primer was based on the MAGI sequence, and the right primer was designed using an alignment of the MAGI and FST. Second, a single right primer was designed when the MAGI showed partial overlap with the FST. These primers were designed using either an alignment between the FST and MAGI or were designed with the FST alone. Third, a presumptive right primer was designed for the 15 FSTs that did not have MuTIR sequences based on the hypothesis that these MuTAIL products were not completely sequenced.

**Figure 2:**
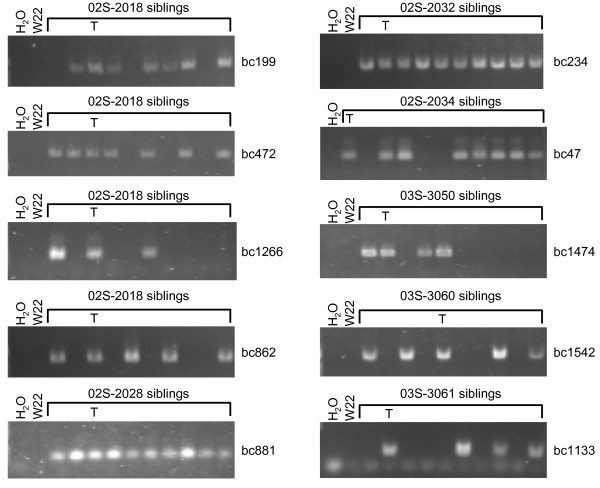
**Examples of PCR validation assays for UniformMu FSTs**. Each panel shows PCR products that were amplified using a MuTIR primer and a FST locus-specific primer. Each primer was tested on the initial template DNA used to develop the UniformMu FSTs (lane T) as well as DNA extracted from siblings (denoted by brackets). No DNA template controls were included in all assays (lane H_2_O). W22 DNA was used to confirm that the insertion site did not amplify in the recurrent inbred parent of the UniformMu population.

The locus-specific primers were used in combination with MuTIR primers to amplify the insertion sites from individual mutant lines. The primers were tested with the initial DNA template used to generate the FST, as well as with siblings or progeny of the individuals used for templates. Figure 2 shows examples of PCR validation assays for 10 FSTs. In these examples, the plant used for MuTAIL sequencing came from a backcross of an individual UniformMu line to the W22 inbred. Germinal insertions are expected to be found in all siblings or to segregate in a 1:1 ratio, depending on whether the progenitor plant was homozygous or heterozygous for individual insertion sites. Overall, we confirmed 94 insertion sites from the sample of 106 FSTs, using an average of 1.6 locus-specific primers per FST (Additional file 2). In many cases, two primers for an individual FST were tested simultaneously and both of these primers amplified the *Mu *insertion site (see Additional file 2). For 84 of the insertion sites, a single primer was sufficient to confirm and track the insertion. Importantly, all 94 *Mu *insertions confirmed in these assays showed germinal segregation. Overall, our data indicate that the vast majority of these sequence-indexed mutants are simple to recover from the UniformMu population.

There were two noticeable trends in the FSTs that failed to amplify in the PCR assays (Table 2). First, the locus-specific primers for five insertion sites (bc41, bc182, bc1042, bc1509, and 03S0467-14) were not designed to anneal at the insertion site. Three of these FSTs included repetitive or chimeric sequences that identified MAGI sequences with only a partial sequence match to the FST. In these cases, the specific primers were designed from MAGI sequences that were not part of the FST. For bc182 and bc1042, the locus-specific primers were designed to amplify from the non-MuTIR end of the insertion site sequences. The second trend was observed for a group of four other FSTs (bc1073, bc1382, bc1435, and bc1537). The locus-specific primers for these insertions showed base pair differences between the most recent MAGI or FST consensus sequence suggesting that the primers were originally designed from lower quality sequence reads or assemblies. *Mu*-specific products were not observed from a final group of three insertions sites (bc93, bc1571, and bc1526) for unknown reasons. Overall, 75% of the insertion sites that failed to be confirmed had errors in the locus-specific primer design, suggesting that sequence errors or human errors were the primary causes for difficulties in confirming an insertion site.

**Figure 3:**
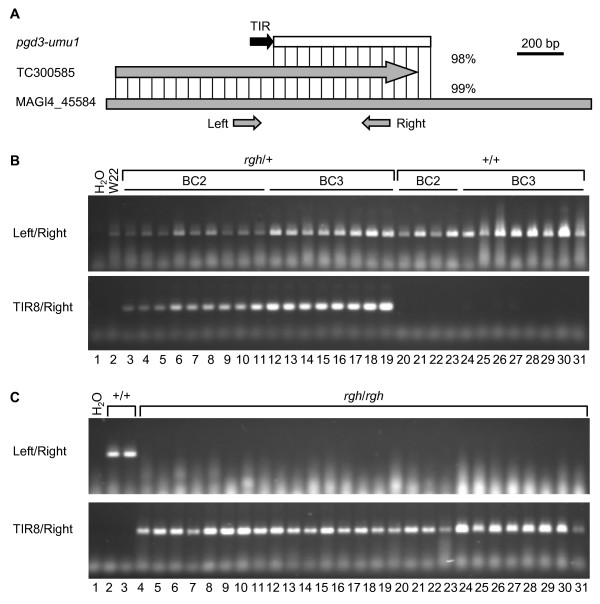
Co-segregation of *pgd3-umu1 *with rgh*-00S-005-14. (**A**) Schematic alignment of the bc199 UniformMu FST site (*pgd3-umu1*), the TC300585 Zea mays Gene Index EST contig, and the maize assembled genomic island, MAGI4_45584. The schematic shows the location of the MuTIR sequence (black arrow, not drawn to scale) and percent nucleotide identity is noted. The left and right primer sites are marked by gray arrows; primer sizes are not drawn to scale. (**B**) Co-segregation analysis with progeny from backcross generations 2 and 3 (BC2 and BC3) in a W22 introgression. Left/Right products amplify normal alleles. TIR8/Right products amplify the insertion site. (**C**) Co-segregation analysis with self progeny. PCR was completed with DNA extracted from homozygous normal (lanes 2–3) or *rgh *mutants kernels (lanes 4–31).

The PCR validation assays also identified an insertion site, bc199, that co-segregates with a *rough endosperm *(*rgh*) seed mutant phenotype. The bc199 insertion disrupts the open reading frame of a predicted 6-phosphogluconate dehydrogenase (6PGDH) gene at the *Pgd3 *locus, and we named this insertion allele *pgd3-umu1*. There are three known loci in maize for 6PGDH enzymes: *Pgd1*, *Pgd2*, and *Pgd3 *[29]. *Pgd3 *maps to the short arm of chromosome 4 in bin 4.03 [30]. We designed both left and right locus-specific primers to develop a co-dominant PCR assay for this insertion (Figure 3A). Co-segregation between the *pgd3-umu1 *insertion site and the rgh*-00S-005-14 mutant was observed in a test of 29 progeny from backcrosses to W22 (Figure 3B) as well as 28 homozygous *rgh/rgh *mutant individuals from a segregating self-pollination (Figure 3C). We expanded the linkage analysis to test a total of 260 meiotic products and observed no recombination between *pgd3-umu1 *and the *rgh *mutant (data not shown). These data indicate that the map distance between *pgd3-umu1 *and the *rgh *phenotype is less than 0.4 cM. This tight linkage raises the hypothesis that the *rgh *mutant phenotype may represent the *pgd3-umu1 *knockout phenotype, and we characterized the rgh*-00S-005-14 mutant further.

**Figure 4:**
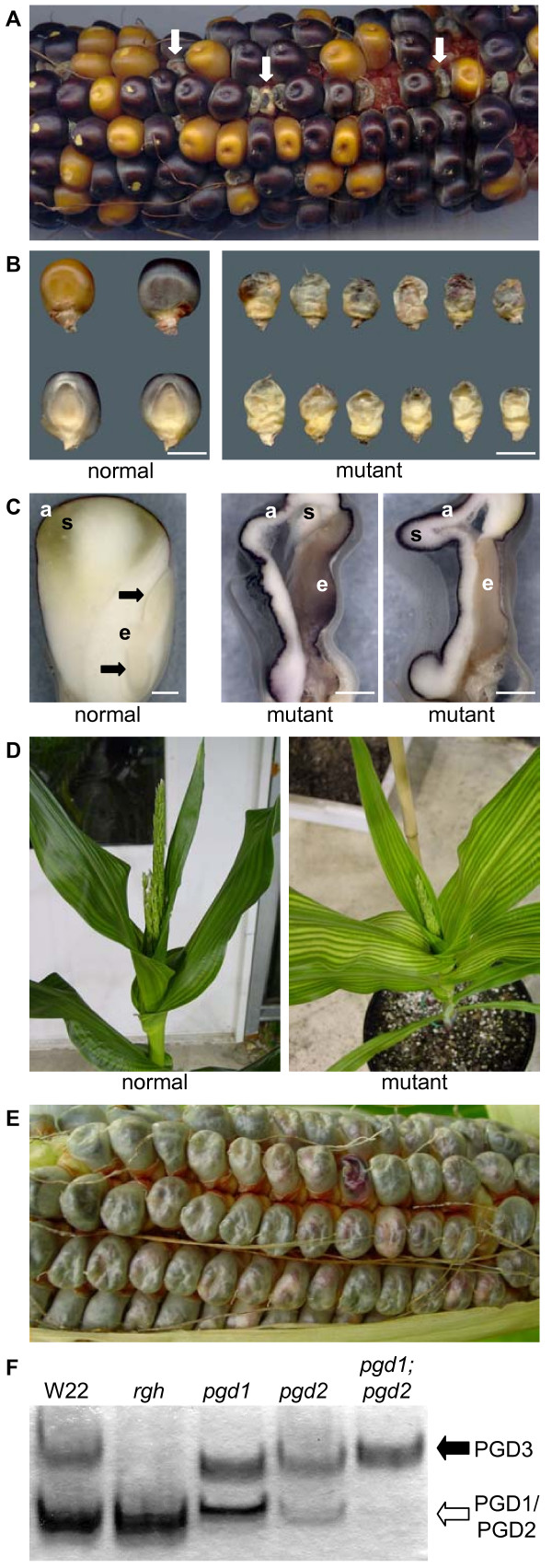
Phenotype of the rgh*-00S-005-14. (**A**) Self-pollinated ear segregating for *rgh *kernels (arrows). (**B**) Mature normal and *rgh *sibling kernels. The top row shows the abgerminal side of the kernels, and the bottom row shows the germinal side of the kernels. Scale bars are 5 mm. (**C**) Longitudinal hand sections of normal and *rgh *kernels. Aleurone, starchy endosperm, and embryo tissues are denoted by a, s, and e, respectively. Arrows indicate embryonic shoot and root tissues in the normal kernel. Scale bars are 1 mm. (**D**) Normal and *rgh *mutant plants 50 days after initiating tissue culture. (**E**) Homozygous *rgh *mutant ear at 35 DAP. (**F**) Native PAGE assay for 6PGDH activity from 25 DAP endosperm extracts. Black arrow indicates the inferred PGD3 isozyme and the open arrow indicates the PGD1 and PGD2 homo- and heterodimer isozymes.

The linked *rgh *mutant shows highly reduced grain-fill, along with a characteristic etching or pitting at the endosperm surface (Figure 4A–B). Longitudinal hand sections of mature mutant kernels showed both reduced endosperm development as well as embryo defects, with most mutants failing to develop embryonic roots or leaves (Figure 4C). Rare embryos with a visually apparent shoot-root axis were observed in homozygous mutants. A small fraction of these seeds were able to germinate when cultured prior to seed desiccation. These mutant escapes developed into morphologically normal seedlings and plants (Figure 4D). However, the plants showed a pale green leaf phenotype that was less severe near leaf veins. The homozygous *rgh *individuals were also confirmed as homozygous for the *pgd3-umu1 *allele using the locus-specific PCR assay (data not shown). Self or sibling pollinations of these mutant plants showed all mutant seed, which confirmed that the plants were homozygous *rgh *individuals and not the result of hetero-fertilization (Figure 4E).

6PGDH enzyme activity was measured from 25 days after pollination (DAP) endosperm extracts of W22, rgh*-00S-005-14 homozygous mutants as well as single and double *pgd1*; *pgd2 *mutants (Figure 4F). The W22 sample showed two major isozyme activities. The faster migrating activity corresponds to three bands: PGD1 homodimers, PGD1/PGD2 heterodimers, and PGD2 homodimers [31]. A subset of these bands are absent in the *pgd1 *and *pgd2 *single mutants, and all three bands are lost in *pgd1; pgd2 *double mutant samples. The *rgh *mutant sample lacks the slower migrating activity. Serial dilutions of the W22 extract suggest that the *rgh *mutant endosperm has <5% normal activity for this 6PGDH isozyme (data not shown). We conclude the slower migrating 6PGDH isozyme is likely to be PGD3 as no reduction of this isozyme activity was observed in the other *pgd *mutants. These data suggest that the FST for *pgd3-umu1 *identified an enzymatic knockout.

**Figure 5:**
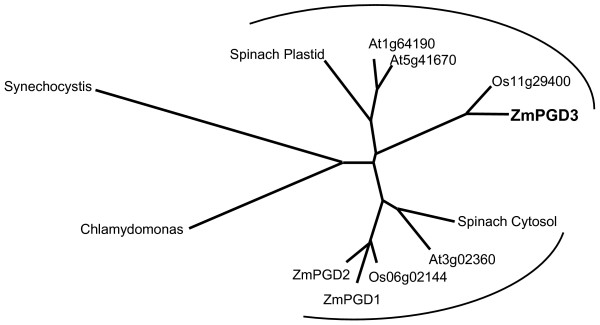
**Sequence similarity tree of 6PGDH enzymes**. Sequence similarity tree of 6PGDH enzymes from maize (Zm), rice (Os), *Arabidopsis *(At), spinach, *Chlamydomonas reinhardtii*, and *Synechocystis *sp. PCC 6803. Rice and *Arabidopsis *proteins are identified by their locus numbers. The protein sequences used to generate the ClustalW alignment and tree were from the ORF of the *Pgd3 *locus and Genbank accessions: AAC27702, AAC27703, AAK49897, AAK51690, AAL76323, AAO42814, ABA93694, NP_198982, NP_442035, NP_850502, NP_910282.

6PGDH enzymes are found in both the cytosol and chloroplast in plants (reviewed in [32]). To predict the likely subcellular localization of the PGD3 protein, a complete *Pgd3 *locus was assembled from EST and MAGI sequences. The predicted peptide for the *Pgd3 *locus contains an N-terminal extension that is absent from the predicted PGD1 and PGD2 proteins in a multiple sequence alignment (data not shown). PGD3 clusters with a known chloroplast-localized 6PGDH protein from spinach in a ClustalW sequence similarity tree ([33,34]; Figure 5). Furthermore, the PGD3 protein is predicted to be targeted to the chloroplast by the protein localization programs: TargetP, PSORT, and Predotar [35-37]. These sequence analyses suggest that the *pgd3-umu1 *allele disrupts a chloroplast-localized 6PGDH in maize. This prediction is consistent with previous observations that the remaining 6PGDH activity in *pgd1; pgd2 *double mutants is entirely plastid-localized [29].

## Discussion

The current maize knockout resources have enough insertions to disrupt every gene within the genome. FSTs will make these maize mutants far more available to the research community, and sequencing MuTAIL products is a high throughput approach to generate maize FSTs. However, FSTs alone do not make a reverse genetics resource easy to use. The tags need to have low entry barriers for tracking an insertion site of interest as well as for obtaining the line or strain that corresponds to the insertion site. FSTs from the UniformMu population are predicted to have low entry barriers due to the genetic markers included in the population and the high-throughput sequencing strategy used to generate the FSTs [19].

The *bz1-mum9 *mutant was used to select against somatic activity, and here we have shown that the vast majority of the resulting FSTs segregate as germinal insertions. We did not observe any evidence of somatic insertions. Somatic insertions would be expected to amplify from the DNA template used to generate the FSTs, but not to amplify from any other related individuals. In the cases where we could not confirm an insertion, the insertion site did not amplify from the template DNA used to generate the FSTs.

A secondary analysis of the specific primers indicated that three factors led to a small fraction of insertion sites that are more difficult to track. First, differences between available maize genomic sequences and the FSTs caused the precise insertion site to be mis-identified for 6.7% of the FSTs. These mis-identifications led to errors in locus-specific primer design. The sequence differences are likely to result from sequence errors, incomplete maize genomic sequence, and sequence divergence between the W22 inbred background used to develop UniformMu and the B73 inbred chosen for genomic sequencing. Comparative genomic sequence studies of maize inbreds suggests that there are significant differences in gene content and repetitive elements between inbreds [38-40]. A second factor that contributed to insertion sites that were not confirmed as germinal was human error in identifying the TIR-genomic DNA junction. These errors led to primer design errors for 1.9% of the FSTs. Finally, PCR or sample tracking errors occur at a low frequency in any high-throughput sequencing project. We speculate that a small amount of cross-contamination between sequencing libraries can explain the remaining 2.8% of insertion sites that were not confirmed. Importantly, 89% of the FSTs were readily confirmed as germinal insertions by locus-specific PCR. Furthermore, the MuTAIL FSTs were generated from individual plants or lines that were successfully bulked, and each sequence read name corresponds directly to an individual. These characteristics make it simple to identify the seed stocks for each FST. Combined, the characteristics of the MuTAIL FSTs and the UniformMu population make a robust resource for reverse genetics.

The *pgd3-umu1 *insertion site that co-segregates with a seed mutant phenotype illustrates the utility of the UniformMu FST resource for generating hypotheses about gene function. The *pgd3-umu1 *insertion is in a predicted chloroplast-localized 6PGDH enzyme in the oxidative section of the pentose phosphate pathway (OPPP). The linked rgh*-00S-005-14 seed mutant lacks a 6PGDH isozyme activity suggesting *pgd3-umu1 *causes an enzymatic knockout. These linkage and biochemical data suggest the hypothesis that *Pgd3 *has an essential role in seed development. Consistent with this hypothesis, the OPPP has been shown to be involved in hexose cycling and starch synthesis in developing seeds [41]. Also, previous studies with maize *pgd1 *and *pgd2 *mutants argued that the plastidic enzymes for the oxidative section of the OPPP can compensate for loss of 6PGDH in the cytosol [29,31]. Moreover, a study in maize root tips found that the oxidative section of the OPPP to be most active in plastids [42]. Combined these data are consistent with plastidic OPPP enzymes being required for seed development. However, linkage data alone cannot prove that the *rgh *phenotype is a result of the *pgd3-umu1 *mutation. Additional experiments are necessary to test this hypothesis such as identifying multiple mutant alleles of *Pgd3*. Alternatively, a *Pgd3 *transgene could be developed to test for complementation of the 6PGDH enzyme defect and the rgh*-00S-005-14 phenotype. The maize genetics community has multiple resources to assist in identifying alleles or in generating transgenics for individual genes of interest (reviewed in [15]).

The *pgd3-umu1 *example also illustrates the complementary nature of having reverse genetics resources in multiple plant species. The *Arabidopsis *genome contains two copies of genes predicted to encode plastid-localized 6PGDH enzymes and one gene predicted to encode a cytosolic 6PGDH (Figure 5; [32]). Each of these genes have multiple sequence-indexed mutations that disrupt exon sequences and are likely to cause null alleles [43]. Gene redundancy in *Arabidopsis *may be one reason why mutant phenotypes associated with plastid-localized 6PGDH have not been reported previously. If plastidic 6PGDH enzymes are essential in plants, a double mutant for knockouts of Arabidopsis orthologs, At1g64190 and At5g41670, would be expected to show a visible phenotype.

The rice genome has a single locus that is predicted to encode plastid-localized 6PGDH, Os11g29400, similar to maize. However, the current rice knockout resources do not have FSTs that disrupt this locus [44]. The rice ortholog for cytosolic 6PGDH, Os06g02144, has three insertions, but these insertions map to promoter and 3' UTR regions and may not be null alleles. Thus, the maize mutants in 6PGDH enzymes suggest an agricultural relevance for the plastid-localized OPPP that would not be as simple to identify using the current *Arabidopsis *or rice resources.

## Conclusion

The *pgd3-umu1 *allele is just one example of the current collection of 1,882 non-redundant insertion sites sequenced in UniformMu. We conclude from our analysis that the vast majority of the UniformMu insertion sites are germinal. Importantly, multiple studies have shown that *Mu *insertions are heavily biased for transcribed regions of the genome [17,19,22,23]. In addition, the UniformMu population is in an inbred background that simplifies phenotypic analysis [19]. Combined, these features provide a robust, genome-wide resource for reverse genetics in maize. Further sequencing of UniformMu FSTs would enhance this resource by expanding it towards a comprehensive knockout collection.

## Methods

### Sequence Analysis

The UniformMu FSTs were clustered onto maize sequences using the methods described in McCarty et al. [19]. In addition, the FSTs were assembled using CAP3 [26], and the FST contigs corresponding to the blastclusters chosen for PCR analysis were retrieved from the assembly for more detailed annotation. The FST contigs were queried against the MAGI4.0 release of the maize genome survey sequence assembly as well as release 16.0 of the *Zea mays *gene index (ZMGI) using BLASTN searches [27,28,45]. Top matches were visually inspected to determine if the sequence alignment was consistent with a precise match to the maize genome or tentative consensus EST contig. An insertion site was considered precisely identified when the top match had >95% identity between the FST and the MAGI or ZMGI. For matches to MAGI sequences, alignments were expected to extend through the full length of the MAGI or FST sequence with no insertion or deletion polymorphisms >20 bp. When a FST showed significant identity with more than one MAGI, the MAGI sequence that defined the precise insertion site was reported. The MAGI sequence matching each FST was also queried against the ZMGI to identify MAGIs with evidence for a transcribed region.

Each FST was scored for the presence of the MuTIR by visual inspection of the terminal end sequence of the FST contigs. Insertion site positions were classified as follows. Promoter insertions had TIR-genomic DNA junctions that were <850 bp 5' of the ZMGI tentative consensus EST contig. Exon insertions had a TIR junction within a ZMGI sequence. Intron insertions had a TIR junction in genomic DNA that could be positively identified as intron sequence based on an alignment of the FST, MAGI, and ZMGI sequences. Unknown insertions did not have a ZMGI match with either the FST or corresponding MAGI sequence. FSTs without a clear TIR sequence were also classified as unknown.

The *Pgd3 *locus was assembled by aligning the bc199 FST contig with MAGI4_45584, MAGI4_45583, and the ZMGI TC300585 contig. The largest open reading frame (ORF) was selected and used to generate the protein similarity tree with ClustalW and TreeDraw, which were run on the SDSC Biology Workbench [34,46,47].

### PCR Assays

The PCR assays were completed with multiple methods, and no significant difference was observed between approaches. Detailed methods for specific insertion sites can be obtained from the primary contacts given in Additional file 2 for each insertion site and primer. In general, DNA was extracted as described in Settles, et al. [23]. The locus-specific primers were designed with Primer3 for 20–25 base oligomers with a predicted T_m _between 60–65°C [48]. The specific primers were paired with a MuTIR specific primer. MuTIR primers used in this study included TIR4, TIR6, and TIR8 from Settles, et al. [23] as well as the primers 5'-CGCCTCYATTTCGTCGAATCC-3' and 5'-GCCTCCATTTCGTCGAATC-3'. PCR was typically completed with *Taq *DNA polymerase and buffers from either Invitrogen or Promega with either 5% DMSO or 1 M Betaine added to the final reaction. Thermocycling conditions were generally 94°C for 1 minute, 60°C for 1 minute, 72°C for 1 minute for 40 cycles. Annealing temperatures were adjusted when the predicted T_m _of a specific primer was lower than 60°C. Most specific primers gave products in the first set of PCR conditions tested, and a careful study of optimal general methods for amplifying specific insertions sites was not completed.

### rgh*-00S-005-14 Mutant Phenotypic Analysis

The longitudinal hand sections of mutant and normal *rgh *kernels were completed with mature kernels from a self-pollinated heterozygous individual. The kernels were imbibed in water overnight and sectioned with a fresh razor blade and unstained sections were imaged. To recover homozygous mutant plants, *rgh *mutant and normal seed were removed from mature, segregating self-pollinations (30–35 DAP) prior to drying the ears. The seed were surface-sterilized in 70% ethanol for 2 min, transferred to 20% bleach, 0.1% Tween 20 for 15 min, and washed in sterile water four times. The pericarp was removed from the kernels under sterile conditions and transferred to Murashige and Skoog salt and vitamin mixture at pH 5.7 with 0.4% Phytagel and 1% sucrose. The seeds were cultured at 32/25°C (day/night) with a 16-h photoperiod. Germinated seedlings were transferred to soil after 2–4 weeks of culture, and grown in greenhouse conditions with a similar temperature and light regime.

6PGDH enzyme activity was measured by native-PAGE assays essentially as described by Bailey-Serres et al [31]. Briefly, 0.1 g of endosperm tissue was ground in extraction buffer (20 mM Tris, pH 8.0, 4 mM DTT, 5 mM MgCl_2_, 15% (v/v) glycerol, 0.001% Bromophenol blue) at a ratio of 0.2 mL extraction buffer to 0.1 g tissue fresh weight. The protein extracts were cleared prior to gel loading by centrifugation at 10,000× g for 10 min at 4°C. The extracts were loaded based on equal fresh weight of starting samples and the proteins were separated at 4°C with native-PAGE (7% acrylamide, 0.375 M Tris, pH 8.8, 12% glycerol(v/v)). After electrophoresis, the gel was stained for 6PGDH (0.1 M Tris-HCl, pH 7.5, 0.1 mg/mL NADP, 0.1 mg/mL nitro blue tetrazolium, 0.01 mg/mL phenazine methosulfate, 0.5 mg/mL 6-phosphogluconate) at room temperature for 30 min. The gel was de-stained with water prior to drying and digital imaging.

## Authors' contributions

AMS completed germinal PCR segregation analysis including locus-specific primer design for 30 of the insertion sites tested, completed phenotypic analysis of *pgd3-umu1*, integrated the data from the other authors including informatics analysis to annotate the insertion sites, supervised the experiments completed by LL, DSF, EW, and CWT, and wrote the manuscript. DRH, BCT, SPL, JLiu, MS, LL, BAO, DSF, and EW completed germinal PCR segregation analysis including locus specific primer design for 76 of the insertion sites tested. CWT and LL completed the tissue culture experiments, enzyme activity assays, and *pgd3-umu1 *co-segregation analysis. JLai participated in the informatic analysis of the FSTs. CTH, WTA, and JB generated the segregating UniformMu populations. JM supervised the experiments completed by JLai and edited the manuscript. LCH supervised the experiments completed by JB and edited the manuscript. KEK supervised the experiments completed by BAO, CTH, and WTA as well as edited the manuscript. PWB supervised the experiments completed by JLiu and edited the manuscript. BAL supervised the experiments completed by DRH and edited the manuscript. DRM supervised the experiments completed by BCT, SPL, and MS, participated in the informatics analysis of FSTs, and edited the manuscript. All authors read and approved the final manuscript.

## Supplementary Material

Additional File 1 **Annotations of Confirmed Germinal Insertion Sites**. Excel table of all the insertions confirmed in this study including the insertions in Table 1. The Blastcluster identifiers are from the UniformMu website[49], and correspond to one or more FSTs in Genbank. The Insertion site base number is relative to the matching MAGI4.0 sequence. The Insertion type was classified as exon, intron, or unknown based on an alignment of the relevant FST, MAGI and ZMGI sequences. The exact number of bases 5' (negative bases) or 3' (positive bases) are given for insertions that were found to be 5' or 3' of a ZMGI cDNA assembly. Click here for file

Additional File 2 **Locus-specific primers used in this study**. Excel table of the locus-specific primers and germinal segregation results from this study. The Blastcluster identifiers are from the UniformMu website [49], and correspond to the insertion sties in Table 1, Table 2, and Additional file 1. Specific contact information for each primer/insertion site is given. Click here for file

## Figures and Tables

**Table 1:** Annotations of the insertion sites confirmed in Figures 2 and 3[Supplementary-material S1]

Blastcluster	MAGI4.0 hit	Insertion site	ZMGI hit	Insertion type	Annotation/Expression Pattern
bc0047	MAGI4_20201	1097	no hit		No EST support for gene
bc0199	MAGI4_45584	731	TC300585	exon	plastid 6-phosphogluconate dehydrogenase
bc0234	MAGI4_4699	748	TC301994	exon	Mixed tissues
bc0472	No hit		TC300035	exon	Probable prefoldin subunit 2
bc0862	MAGI4_29768	394	no hit		No EST support for gene
bc0881	MAGI4_30440	4515	TC290606	exon	Putative ATP dependent copper transporter
bc1133	MAGI4_155851	3281	TC314694	intron	Putative calmodulin-binding protein
bc1266	MAGI4_43305	8436	TC304662	exon	Putative cytosolic chaperonin delta-subunit
bc1474	MAGI4_118260	731	no hit		No EST support for gene
bc1542	MAGI4_139298	550	TC302939	exon	SAM & endosperm expression

A complete table of confirmed insertions is given in Additional file 1 – Annotations of Confirmed Germinal Insertion Sites. The Blastcluster identifiers are from the UniformMu website [49], and correspond to one or more FSTs in Genbank. The Insertion site base number is relative to the matching MAGI4.0 sequence. The Insertion type was classified as exon or intron based on an alignment of the relevant FST, MAGI and ZMGI sequences.

**Table 2:** Annotations of insertion sites that were not confirmed with PCR assays

Blast Cluster/Read Name	MAGI4.0 hit	Insertion site	ZMGI hit	Insertion type	Annotation/Expression Pattern	Likely cause for failure to amplify
03S-0467-13A2-G01	repeat hit only		DR906457	exon	egg cell expressed	primer design error
bc0041	MAGI4_104	252	no hit		No EST support for gene	primer design error
bc0093	MAGI4_65387	128	DN229926	exon	leaf & SAM expressed	unknown
bc0182	MAGI4_121677	no MuTIR	no hit		No EST support for gene	primer design error
bc1042	MAGI4_128194	1502	no hit		No EST support for gene	primer design error
bc1073	MAGI4_94877	3164	TC303220	exon	TM20/Dek34 gene	low quality sequence
bc1382	repeat hit only	no MuTIR	no hit		Novel maize genomic sequence	low quality sequence
bc1435	MAGI4_132661	no MuTIR	DT943333	unknown	thioesterase superfamily	low quality sequence
bc1509	repeat hit only		no hit		Novel maize genomic sequence	primer design error
bc1526	MAGI4_13062	no MuTIR	TC292893	unknown	26S protease regulatory subunit 7	unknown
bc1537	MAGI4_5849	481	TC291433	intron	cold stressed seedling expression	low quality sequence
bc1571	MAGI4_154704	734	TC282381	-40bp	short-chain dehydrogenase/reductase	unknown
